# Phylogenetic distribution of translational GTPases in bacteria

**DOI:** 10.1186/1471-2164-8-15

**Published:** 2007-01-10

**Authors:** Tõnu Margus, Maido Remm, Tanel Tenson

**Affiliations:** 1 Institute of Molecular and Cell Biology, University of Tartu, Tartu, Estonia; 2 Institute of Technology, University of Tartu, Tartu, Estonia

## Abstract

**Background:**

Translational GTPases are a family of proteins in which GTPase activity is stimulated by the large ribosomal subunit. Conserved sequence features allow members of this family to be identified.

**Results:**

To achieve accurate protein identification and grouping we have developed a method combining searches with Hidden Markov Model profiles and tree based grouping. We found all the genes for translational GTPases in 191 fully sequenced bacterial genomes. The protein sequences were grouped into nine subfamilies.

Analysis of the results shows that three translational GTPases, the translation factors EF-Tu, EF-G and IF2, are present in all organisms examined. In addition, several copies of the genes encoding EF-Tu and EF-G are present in some genomes. In the case of multiple genes for EF-Tu, the gene copies are nearly identical; in the case of multiple EF-G genes, the gene copies have been considerably diverged. The fourth translational GTPase, LepA, the function of which is currently unknown, is also nearly universally conserved in bacteria, being absent from only one organism out of the 191 analyzed. The translation regulator, TypA, is also present in most of the organisms examined, being absent only from bacteria with small genomes.

Surprisingly, some of the well studied translational GTPases are present only in a very small number of bacteria. The translation termination factor RF3 is absent from many groups of bacteria with both small and large genomes. The specialized translation factor for selenocysteine incorporation – SelB – was found in only 39 organisms. Similarly, the tetracycline resistance proteins (Tet) are present only in a small number of species.

Proteins of the CysN/NodQ subfamily have acquired functions in sulfur metabolism and production of signaling molecules. The genes coding for CysN/NodQ proteins were found in 74 genomes. This protein subfamily is not confined to *Proteobacteria*, as suggested previously but present also in many other groups of bacteria.

**Conclusion:**

Four of the translational GTPase subfamilies (IF2, EF-Tu, EF-G and LepA) are represented by at least one member in each bacterium studied, with one exception in LepA. This defines the set of translational GTPases essential for basic cell functions.

## Background

Translational GTPases (trGTPases) are proteins in which the GTPase activity is induced by the large ribosomal subunit [1,2]. Several members of this protein family (EF-G, EF-Tu, IF2 and RF3) bind to an overlapping site on the ribosome [1,3-6]. This conserved region of the large subunit includes part of domain II of 23S RNA (the binding site for the antibiotic thiostreptone), part of domain VI (the sarcin-ricin loop), and proteins L11 and L7/12. This region is responsible for activating the trGTPases [1,2].

The specific sequence features of the trGTPases allow proteins that belong to this family to be identified [7]. In bacteria, the family includes proteins that are considered to belong to the "classical" set of translational GTPases (EF-G, EF-Tu, IF2, RF3), proteins that bind to the ribosome and have auxiliary or unidentified functions (SelB, Tet, LepA, TypA), and a group of proteins that have acquired functions in sulfur metabolism and might have lost their ability to bind to the ribosome (CysN/NodQ). Several additional GTPases with sequences that do not group them into the trGTPase family bind to, or have their activities induced by, the ribosome [8-12]. The GTPase activity of these proteins is not activated by the conserved region described above. The present work focuses on the family of trGTPases ("the classic translation factor family" according to Leipe et al., 2002), so these additional proteins are not included.

It has been shown that many members of this family are nearly ubiquitous in bacteria [13-15]. However, these studies were performed on relatively small datasets because few fully sequenced genomes were available. Moreover, there is confusion in the literature about the members of the core set of trGTPases present in all bacteria. For example, some studies find that LepA is ubiquitous [13-15] but this finding has not been confirmed by others [16]. The number of fully sequenced bacterial genomes is now rapidly increasing and several hundred are available in the databases. This provides a basis for studying the presence of trGTPases in many different organisms. Moreover, no attempts were made in the previous studies to identify the trGTPase subfamilies missing from the organisms under investigation. Careful annotation of these missing trGTPases is essential for understanding the global distribution of this protein family. Therefore, we attempted not only to find as many trGTPases as possible but also to find all the trGTPases in the genomes we studied. This approach allows the presence or absence of genes for particular trGTPases in the genomes to be annotated.

Our study reveals the number of genes in nine subfamilies of ribosome-associated GTPases from 191 fully sequenced bacterial genomes. Four of the subfamilies (IF2, EF-Tu, EF-G and LepA) are represented at least by one member in all bacteria studied (with one exception in the case of LepA, as discussed below). The other subfamilies (Tet, RF3, SelB, TypA, CysN/NodQ) are present only in some bacteria.

## Results

To analyze the gene content of trGTPases in the fully sequenced genomes we needed to group all the trGTPases into subfamilies. This was done in several steps to ensure that all functional genes were detected and properly classified.

### Creating the initial database

**Figure 1:**
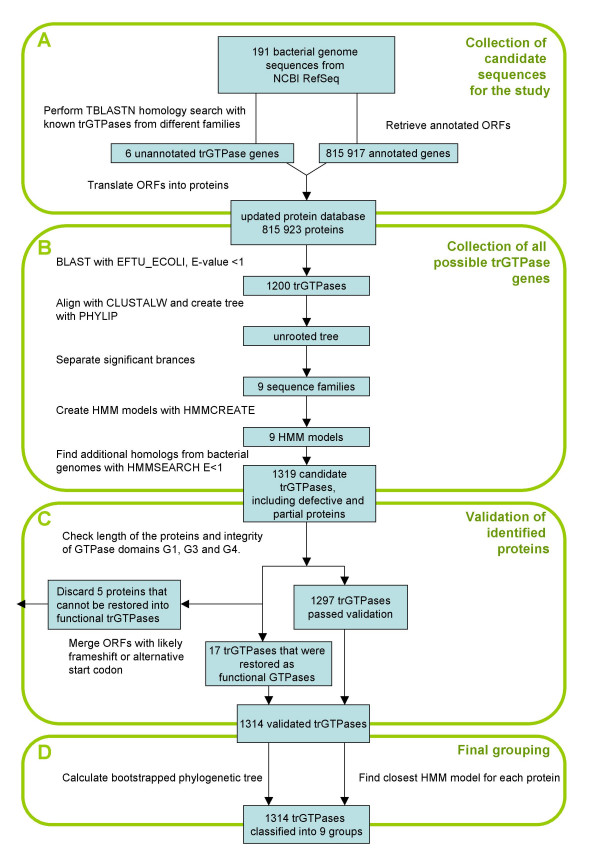
Translational GTPase discovery and grouping flow chart.

We started to gather genes for trGTPases by downloading all the annotated ORF sequences from the RefSeq database [17]. However, this database might contain annotation errors and lack some ORFs. It was important to ensure that none of the trGTPases genes were missing. Therefore, we performed a BLAST search against the genomic sequences using TBLASTN with the nine known trGTPase genes from *Escherichia coli *and the tetracycline resistance gene from *Bacillus cereus*. This search resulted in 6 potential trGTPase genes. Four of these had previously been annotated as pseudogenes, but they could be functional genes. Two additional EF-Tu genes were found (one from *Wolinella succinogenes *and one from *Clostridium acetobutylicum*) that were missing from RefSeq. Interestingly, these two EF-Tu genes were present in GenBank, indicating that RefSeq had missed annotation of these genes. They were added to RefSeq to create a so-called "updated gene database". The ORF sequences in this database were translated into an "updated protein database", which was used for further studies (Fig. 1A).

### Detection of all trGTPase candidates with subfamily-specific HMMs

To ensure that all trGTPases were detected, we used a set of subfamily-specific Hidden Markov Models (HMM) [18]. Subfamily-specific HMMs should detect trGTPase candidates more specifically than the commonly-used BLAST or PSI-BLAST searches. These HMM models were created in several steps: retrieving well-conserved trGTPases from the "updated protein database" by a BLAST search with EF-Tu from *Escherichia coli*, computing a phylogenetic tree, dividing the proteins into nine subfamilies based on the tree and creating subfamily-specific HMMs (Fig. 1B). In addition, "outgroup" HMM profiles were created from 30 non-translational GTPases for control purposes. It is important to notice that the initial tree was calculated using the GTPase domain only, because reliable alignment of full-length sequences is not possible.

All proteins from the "updated protein database" were run against all nine HMM models using HMMSEARCH [18]. Each protein was classified into the most similar family, decided by the HMMSEARCH score. This was done iteratively at increasing sensitivity levels until the number of proteins in all trGTPase families remained unchanged (Table 1); then we retrieved all the potential trGTPases. It is interesting to note that all trGTPases were retrieved at E-value 1e-10, and searches at lower stringency yielded no additional ones (Table 1). The classification of trGTPases was confirmed by calculating a phylogenetic tree as described below ("Final grouping of trGTPases into subfamilies"). It is also important to note that at E-value 1, any of the nine HMM profiles was able to detect members of all other subfamilies. This result indicates that in case there existed an additional trGTPase subfamily, not represented by any of the sequences on our preliminary phylogenetic tree, it would have been detected at this stage.

### Validation of the trGTPases found

The results of the automatic procedures mentioned above were additionally verified by manual inspection (Fig. 1C). Although most of the proteins in our set of trGTPase candidates proved valid, there was also a small subset of proteins that cannot be GTPases because they lack the highly conserved consensus elements (G1, G3 and G4 motifs) of the GTPase domain [16,19]. In addition, four of the proteins were very short (less than 60% of the average protein length of the subfamily). All these cases (listed in Additional file 2 as exceptions) were annotated separately.

**Figure 2:**
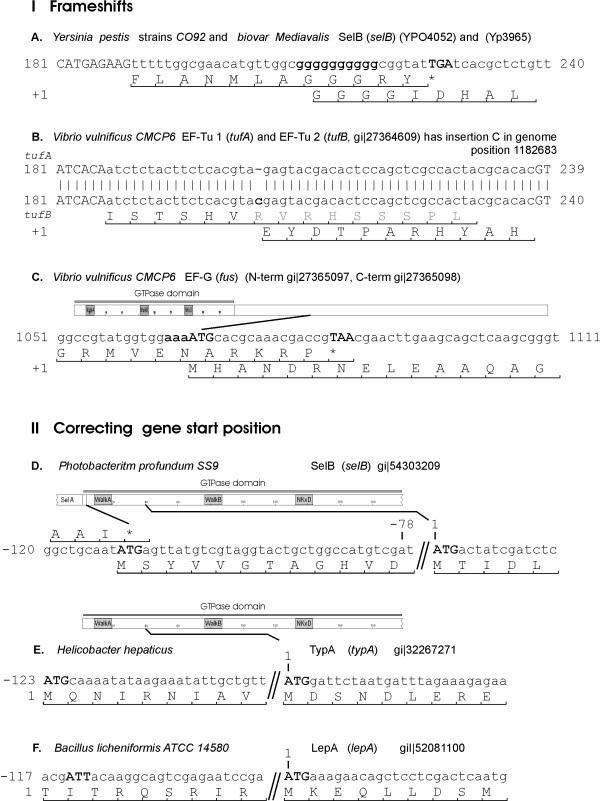
Some examples of frame-shifts (I) and alternative gene start positions (II) for genes marked as exceptions. The full list of exceptions is presented in Additional file 1.

In seven cases there was an upstream start codon that was not annotated as a functional start codon but would allow a functional protein to be produced. For example, in the current annotation, the correct start position is missed in the *Photobacterium profundum *SelB coding gene because it overlaps with the stop codon of the previous gene (*SelA*). In other cases, an alternative, non-AUG initiation codon could restore a functional protein. For example, in *Borrelia burgdorferi *and *Bacillus licheniformis*, full length *lepA *can be restored only if we assume that AUU is a start codon (Fig. 2F and see Additional file 1). There are two existing examples in which AUU has been shown to function as an initiation codon: in *Escherichia coli infC *(coding for IF-3), AUU regulates expression at the translational level [20]; and expression of *pncB *is reduced because of the AUU start codon [21].

In our dataset, there are also cases where a frame-shift event might restore a functional gene. In some of these, frame-shift is a probable case. For example, during translation of *selB *in *Yersinia*, frame-shift might occur at the poly(G)_10 _track. Homopolymeric tracks are known to be frame-shifting sites [22].

In conclusion, we found that in 17 cases a functional protein might be restored (Fig. 2, see Additional file 1). These examples are included in the final list of trGTPases and the correction of initiation site or frame-shift event is indicated in Figs. 4, 5, 6. After manual inspection and validation we ended up with 1314 trGTPase proteins (see Additional file 2). These proteins were classified into 9 different families.

### Final grouping of trGTPases into subfamilies

**Figure 3:**
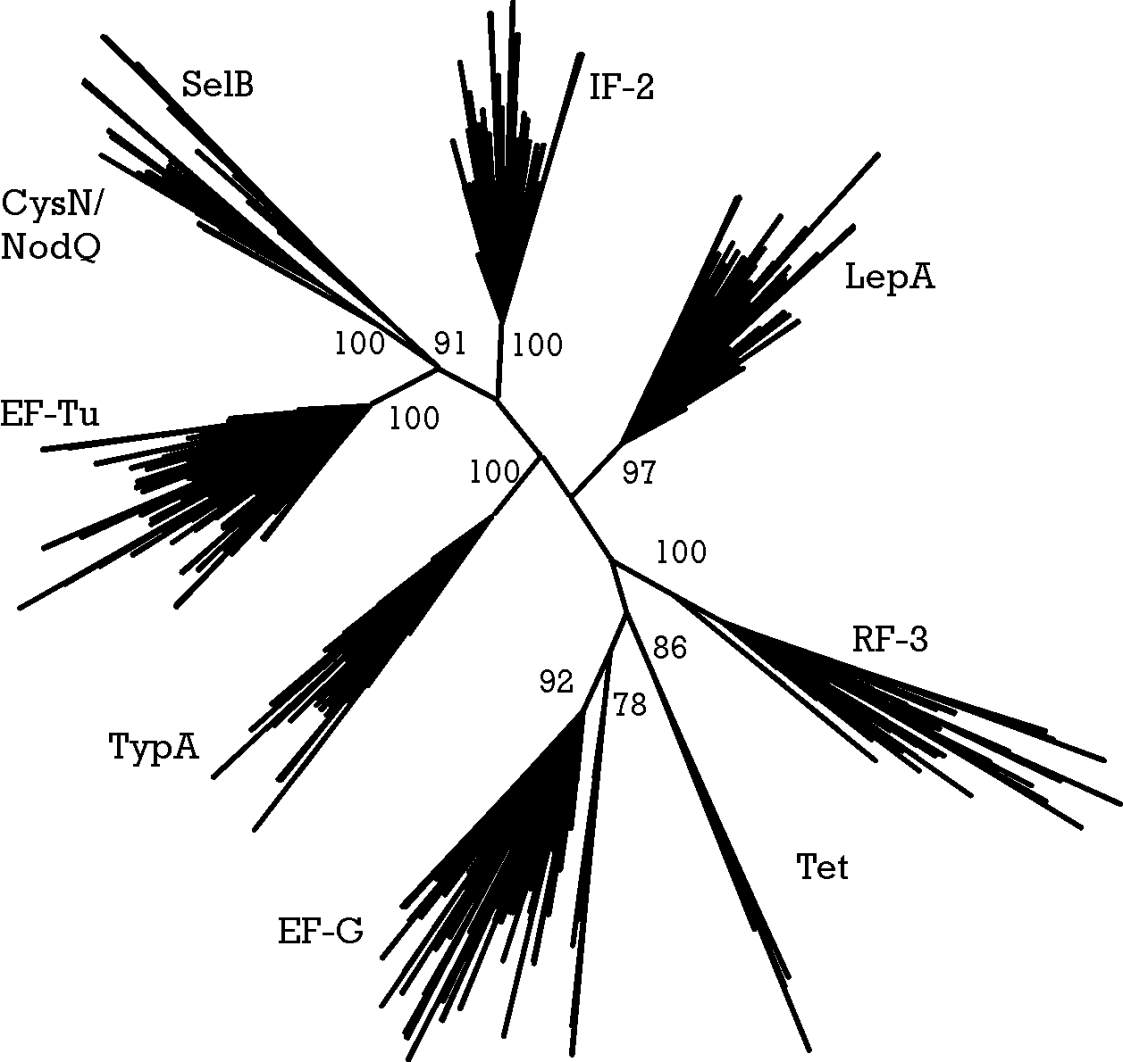
Unrooted consensus tree of translational GTPases. Nine major groups are distinguished by high bootstrap values that are shown by numbers on roots of branches. Underlying multiple alignment is based on GTPase domain alignment made with HMMALIGN [18] against GTP_EFTU model from Pfam database. Tree is calculated using PROTDIST (using JTT matrix) [23], NEIGHBOR and CONSENSE (Extended Majority Rule) from the PHYLIP 3.62 package [23]. One hundred bootstraps were performed to evaluate branch reliability.

The initial grouping of the trGTPases into nine subfamilies was dependent on the initial tree, which was created from a smaller subset of proteins and contained some non-functional proteins. Thus, we decided to confirm the classification of trGTPases again, (a) by dividing proteins among 9 HMMs and (b) by computing a phylogenetic tree from all 1314 validated trGTPases (Fig. 1D). The tree was calculated again using only the GTPase domain, which is universally conserved in all trGTPases. A distance-based phylogenetic tree was created and bootstrapped using PHYLIP [23] with PAM distances (Fig. 3). On this tree the same familiar nine branches appeared with high bootstrap support. Furthermore, all the proteins fell into the same branches as they did using the HMM classification. Thus, the phylogenetic tree supports the classification of proteins into 9 subfamilies as described in Table 1.

There appears to be an additional well-separated branch within the EF-G branch (Fig. 3). However, in quartet puzzling tree (TREE-PUZZLE [24]) and identity-based distance tree, this branch disappears. Therefore, we did not treat it as an independent family of trGTPases in the current study. Nevertheless, this branch may contain EF-G-like proteins that are diverging functionally, as it contains only proteins encoded in genomes with more than one gene for the EF-G subfamily.

**Figure 4:**
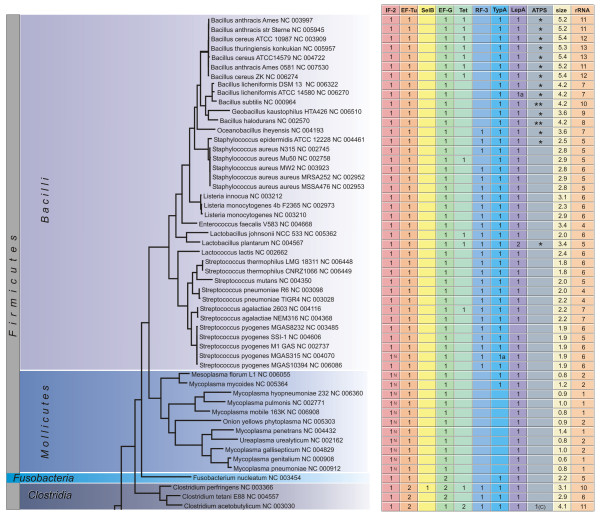
Phylogenetic distribution of translational GTPases. The number of genes in different trGTPase subfamilies is shown in the context of the 16S ribosomal RNA based phylogenetic tree (The bar indicates 0.1 PAM units). The genome sizes in millions of basepairs ("size") and rRNA operon copy numbers ("rRNA") are also shown. The symbol "a" indicates that the gene (or one of the genes, in case of multiple genes) might be translated using an alterative in-frame start codon (Fig. 2, see Additional file 1); the symbol "b" indicates that the gene (or one of the genes, in case of multiple genes) might be translated through a frame-shift event (Fig. 2, see Additional file 1). In the IF2 column the proteins containing only one IF2N domain are marked with "N". In the ATPS column the numbers indicate proteins of the CysN/NodQ subfamily (ATPS2). The CysN ("C") and NodQ ("Q") proteins are shown separately. For example, "1(C)2(Q)" indicates the presence of one CysN and two NodQ proteins. The ATPS1 family is marked with "*" ("**" indicates two proteins of this family).

**Figure 5:**
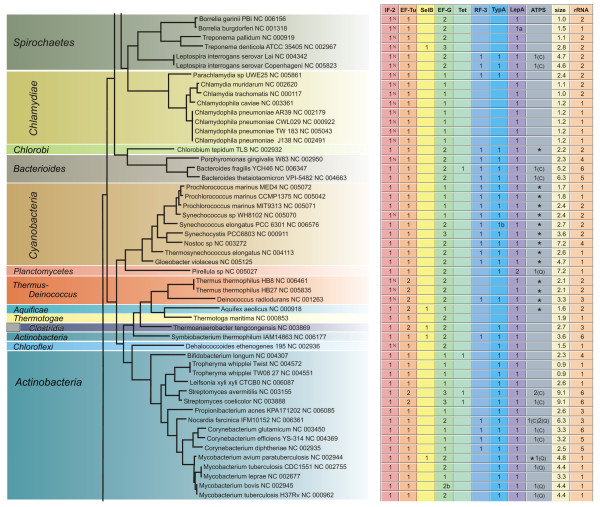
Phylogenetic distribution of translational GTPases. The number of genes in different trGTPase subfamilies is shown in the context of the 16S ribosomal RNA based phylogenetic tree (The bar indicates 0.1 PAM units). The genome sizes in millions of basepairs ("size") and rRNA operon copy numbers ("rRNA") are also shown. The symbol "a" indicates that the gene (or one of the genes, in case of multiple genes) might be translated using an alterative in-frame start codon (Fig. 2, see Additional file 1); the symbol "b" indicates that the gene (or one of the genes, in case of multiple genes) might be translated through a frame-shift event (Fig. 2, see Additional file 1). In the IF2 column the proteins containing only one IF2N domain are marked with "N". In the ATPS column the numbers indicate proteins of the CysN/NodQ subfamily (ATPS2). The CysN ("C") and NodQ ("Q") proteins are shown separately. For example, "1(C)2(Q)" indicates the presence of one CysN and two NodQ proteins. The ATPS1 family is marked with "*" ("**" indicates two proteins of this family).

**Figure 6:**
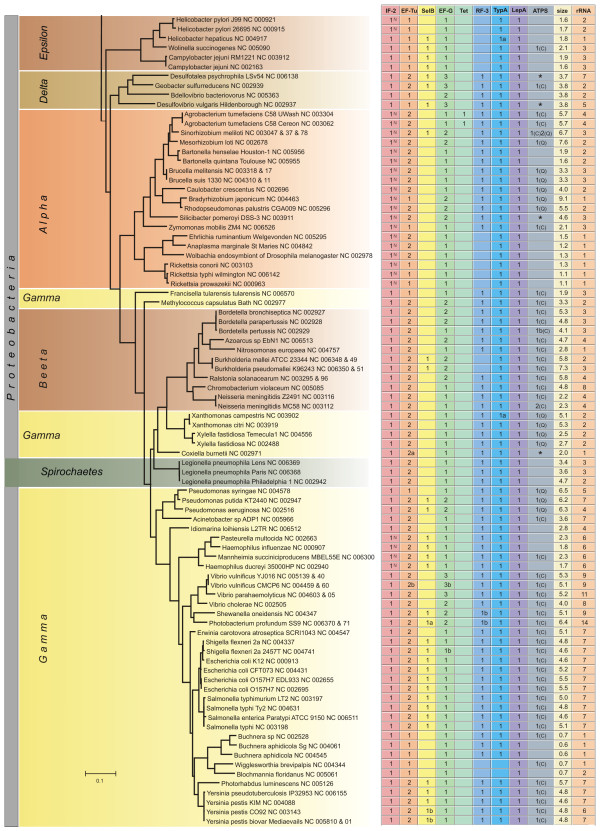
Phylogenetic distribution of translational GTPases. The number of genes in different trGTPase subfamilies is shown in the context of the 16S ribosomal RNA based phylogenetic tree (The bar indicates 0.1 PAM units). The genome sizes in millions of basepairs ("size") and rRNA operon copy numbers ("rRNA") are also shown. The symbol "a" indicates that the gene (or one of the genes, in case of multiple genes) might be translated using an alterative in-frame start codon (Fig. 2, see Additional file 1); the symbol "b" indicates that the gene (or one of the genes, in case of multiple genes) might be translated through a frame-shift event (Fig. 2, see Additional file 1). In the IF2 column the proteins containing only one IF2N domain are marked with "N". In the ATPS column the numbers indicate proteins of the CysN/NodQ subfamily (ATPS2). The CysN ("C") and NodQ ("Q") proteins are shown separately. For example, "1(C)2(Q)" indicates the presence of one CysN and two NodQ proteins. The ATPS1 family is marked with "*" ("**" indicates two proteins of this family).

**Figure 7:**
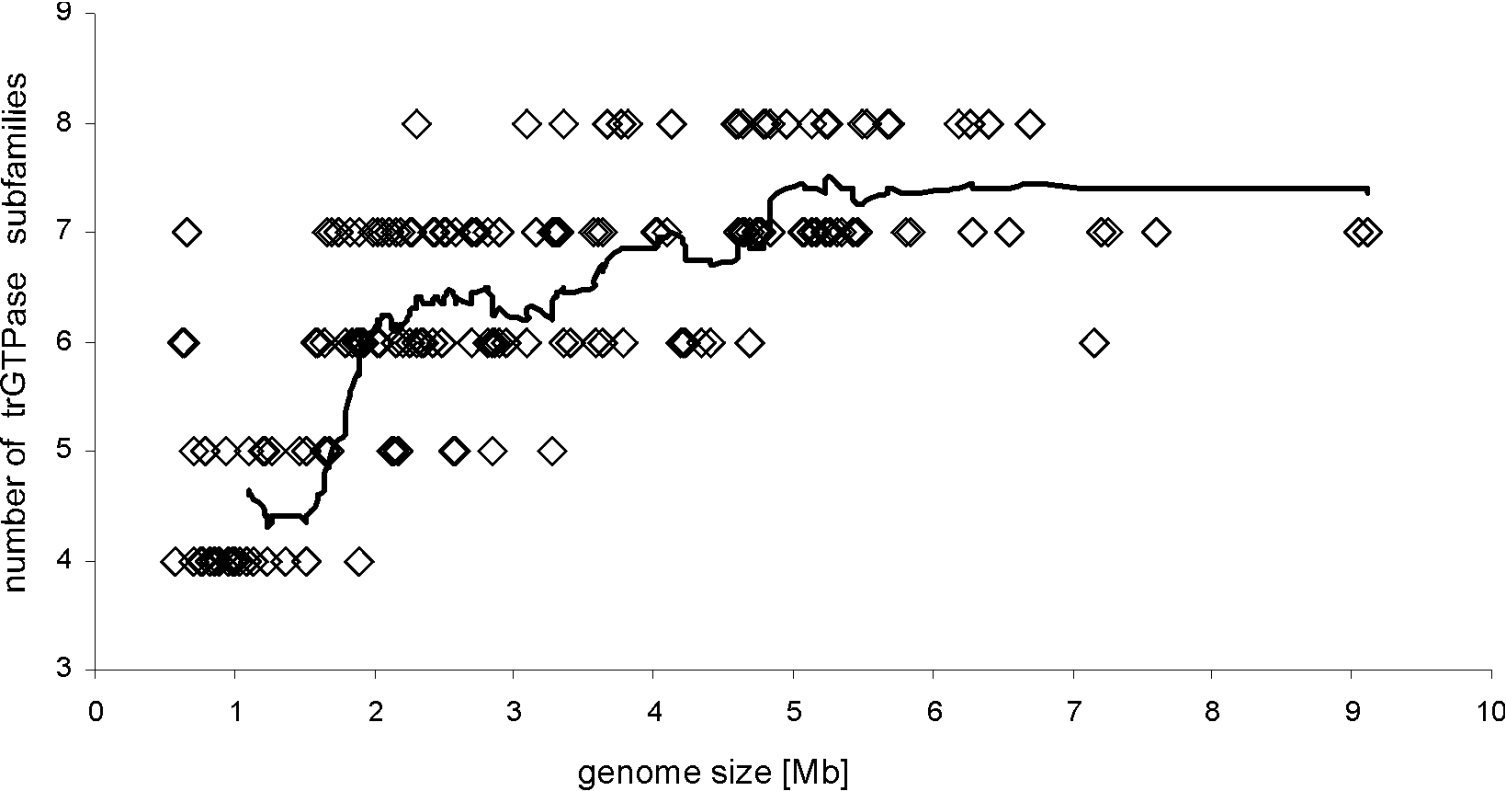
The number of trGTPase subfamilies encoded in one genome presented in correlation with genome size. A sliding window with length 15 genomes was used to draw the trendline.

After identifying all the genes for trGTPases in the genomes under study, we considered the presence or absence of these genes in different phylogenetic groups of bacteria. The number of genes for each trGTPase subfamily is presented in Figs. 4, 5, 6. The 16S ribosomal RNA tree and the phyla of Bergey's bacterial systematics [25] are also shown. We analyzed the relation between genome size (Figs. 4, 5, 6, "size") and the number of trGTPase subfamilies it codes for (Fig. 7). Smaller genomes clearly contain fewer genes for trGTPases. Many small genomes (shorter than 2 Mb) code only for the core set of four trGTPases (IF2, EF-Tu, EF-G and LepA). As the genome size increases, the number of different trGTPase genes also increases, reaching a plateau value between 7 and 8 genes. There are some notable exceptions: the *Buchnera *genomes, which are only 0.6–0.7 Mb, contain 6–7 trGTPase genes; *Pirellula *with genome size 7.2 Mb codes for only six subfamilies of trGTPases, lacking the gene for RF3.

## Discussion

We have annotated the genes for trGTPases in 191 fully sequenced bacterial genomes. The approach we have developed (Fig. 1) allows misannotations, possible sequencing errors, frameshifts and non-canonical translation initiation events to be identified (Fig. 2). We paid special attention to finding all the trGTPases genes in the genomes analyzed. This allows cases where certain subfamilies are not encoded in a given genome to be annotated with confidence.

**Figure 8:**
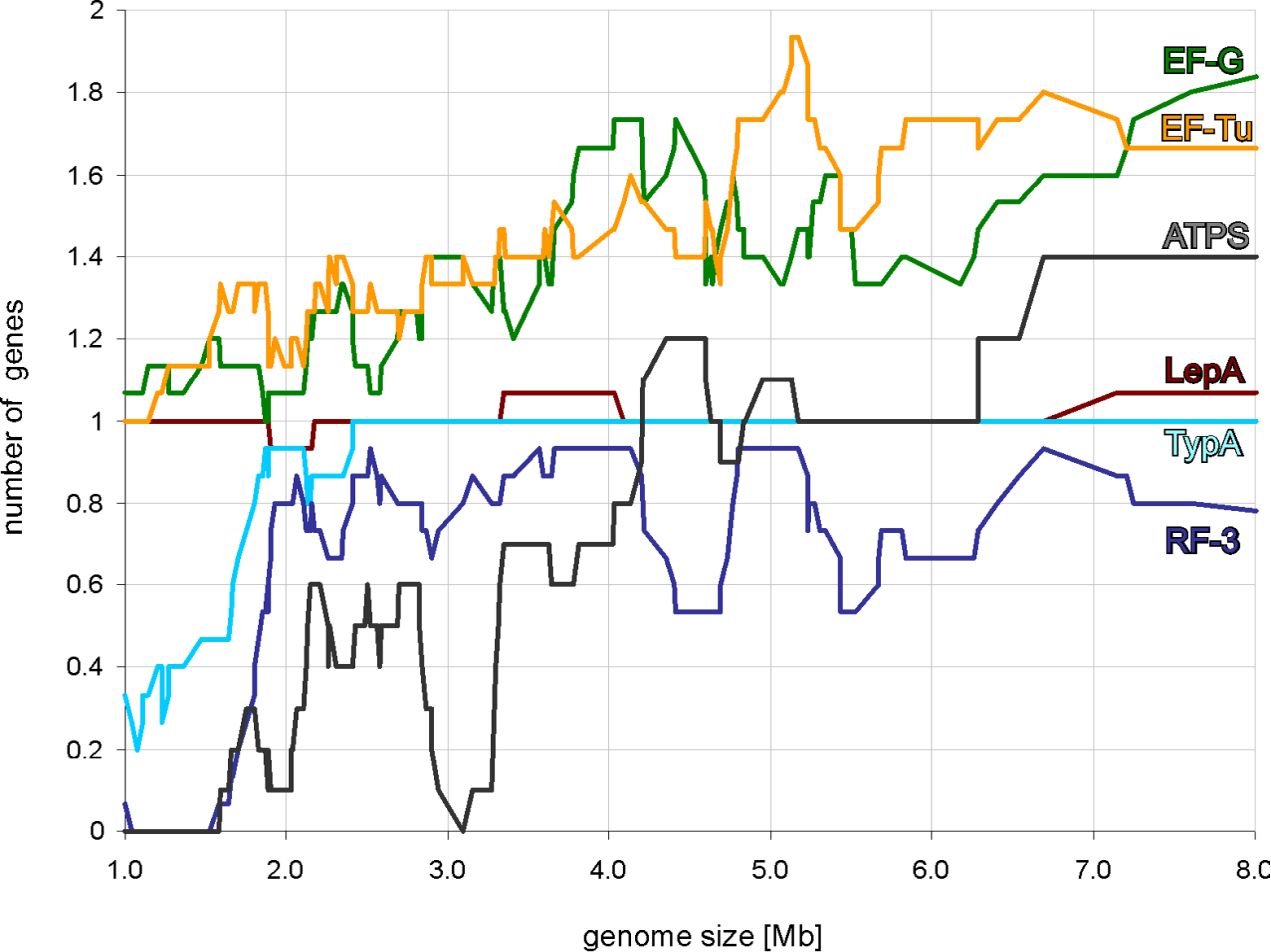
The number of gene copies in each subfamily presented in correlation with genome size. The ATPS proteins include both CysN/NodQ (ATPS2) and ATPS1. A sliding window with length 15 genomes was used to draw the trendline.

Our study reveals the number of members in nine subfamilies of ribosome-associated GTPases. Four of the subfamilies (IF2, EF-Tu, EF-G and LepA) are represented by at least one member in each bacterium studied, with one exception in LepA, as discussed below (Figs. 7, 8). The other subfamilies (Tet, RF3, SelB, TypA, CysN/NodQ) are present only in some bacteria (Figs. 4, 5, 6). In the following sections the trGTPases subfamilies are discussed in detail.

### Initiation factor 2

The bacterial IF2 catalyzes the binding of initiator tRNA to the initiating 30S subunit [26,27]. In the next step the GTP-bound IF2 catalyzes formation of the 70S ribosome [28,29]. Ribosome-stimulated GTP hydrolysis is required for rapid dissociation of the factor from the ribosome [29].

The gene for IF2 was recognized in all the genomes analyzed. This indicates that IF2 is absolutely conserved in all bacteria (Figs. 4, 5, 6). Moreover, previous analysis has identified the gene for IF2 as universally conserved in all domains of life [30,31]. It is also consistent with the fact that deletion of the gene for IF2 is lethal in *Escherichia coli *[32]. In contrast to several other ribosome-associated GTPases described below, the gene for IF2 has not been duplicated in any of the genomes analyzed; all bacteria contain only one copy.

*Escherichia coli*, other members of the family *Enterobacteriaceae *and *Bacillus subtilis *all contain two or three isoforms of IF2, resulting from the use of different in-frame start codons [33-35]. Both the longer and shorter isoforms contain the major functional domains of the protein, including the GTPase domain, and are functionally active in biochemical assays [29,36]. However, an optimal ratio of isoforms is required to achieve maximal growth rate [37,38]. A conserved domain (IF2N) has been described in the N-terminus of the protein [39]. In *Escherichia coli *the longer isoform contains two copies of the IF2N domain and the shorter isoforms have one copy. In our collection of IF2 sequences the tandem organization of the IF2N domain was found in 134 cases out of the 191 analyzed. This suggests that these proteins are annotated as the longer isoforms. Although the presence of IF2 isoforms has been experimentally proven in several organisms [33-35], an experimental study using a wider phylogenetic range of bacteria is needed to clarify the generality of an internal initiation event occurring between the two IF2N domains. In 57 IF2 sequences, only one IF2N domain was found (marked with symbol "N" in Figs. 4, 5, 6). This suggests that in these organisms only the shorter isoform of IF2 is present.

### Elongation factor Tu

EF-Tu in complex with GTP brings aminoacyl-tRNA into the A site of the ribosome [2]. The factor is released from the ribosome after GTP hydrolysis [40]. GTP hydrolysis separates two steps in the selection of the correct codon-anticodon interaction: initial selection occurs before hydrolysis and proofreading occurs afterwards [2,41]. This double-stage selection of aminoacyl-tRNA allows the accuracy of translation to be increased [41-43]. Exchange of EF-Tu-bound GDP with GTP relies on a specific G-nucleotide exchange factor, EF-Ts [44-46].

We found the gene for EF-Tu in all genomes analyzed (Figs. 4, 5, 6, 8). This agrees with the previous notion that this trGTPase is universally conserved in all three domains of life [13,14]. In our dataset, 267 proteins (from 191 organisms) belong to the EF-Tu family, encoded in 1 to 2 copies of the gene per genome. Most of the bacteria with two EF-Tu genes belong to the phylum *Proteobacteria *(45 species), but there are also additional genes in *Firmicutes *(class *clostridia*) (3), *Deinococcus-Thermus *(2), *Actinobacteria *(2) and *Aquificae *(1). For *Proteobacteria*, it has been argued that the observed phylogenetic distribution is best accounted for by the presence of two gene copies in the ancestral genome followed by differential loss of the second copy [47].

The function of EF-Tu is essential for the cell and its gene cannot be deleted [48]. In *Escherichia coli*, where two EF-Tu-coding genes are present, either of them may be deleted without affecting the viability of the cell. Interestingly, if the organism has two copies of the EF-Tu gene, then the two copies are nearly identical. Gene conversion is assumed to be the mechanism behind this similarity. This was proved to be the case in *Salmonella typhimurium *[49-51]. A similar mechanism maintains the uniformity of sequences of different ribosomal RNA operons in some genomes [52-54].

The genomes analyzed in the current study contain between 1 and 14 ribosomal RNA operons per genome. The larger number of ribosomal RNA operons might indicate the need for more ribosomes and other components of the translational machinery, including EF-Tu. We therefore asked whether there are more rRNA operons in genomes containing two gene copies for EF-Tu than in those of bacteria with only one EF-Tu-coding gene (Figs. 4, 5, 6; data not shown). No clear correlation can be found because there are genomes with many rRNA operons and one EF-Tu gene copy (*Bacillus*), and genomes with only one rRNA operon and two EF-Tu gene copies (*Ehrlichia, Anaplasma, Wolbachia, Nitrosomonas*). The EF-Tu gene copy number rather follows the phylogenic clades: most of the *Proteobacteria *have two and most of the other phylogenetic groups have one.

### Elongation factor G

EF-G catalyzes the translocation of peptidyl-tRNA from the ribosomal A site to the P site and of deaminoacylated tRNA from the P site to the E site [2,55]. The exact mechanism by which GTP is used in this process is currently under discussion [56-60]. In addition to its role in translocation, EF-G is required to recycle the ribosomes from their post-termination state to a new round of initiation [61-64].

Consistent with the observation that EF-G is the third trGTPase universally conserved in all three domains of life [13,14], we found the gene in all the genomes analyzed (Figs. 4, 5, 6, 8). In the model organisms *Escherichia coli *and *Bacillus subtili *EF-G is encoded by one essential gene. Surprisingly, we found that in 47 of the 191 genomes analyzed there are two genes for proteins of the EF-G subfamily, and in 10 genomes there are three copies (Figs. 4, 5, 64-6, 8). Multiple gene copies for EF-G are found widely in the bacterial phylogenetic tree, being observed in most of the phyla analyzed.

In contrast to EF-Tu, the copies EF-G genes in one genome differ considerably; the gene conversion mechanisms that work in case of the EF-Tu coding genes do not seem to operate in the case of EF-G. It is currently not clear whether the two copies of EF-G are functionally similar or whether one form might have acquired a different function.

### Tet proteins

In one case we know that a separate group of ribosome-associated GTPases has evolved from the EF-G subfamily. This is the subfamily of tetracycline resistance proteins [65,66]. In the current work we use the abbreviation "Tet" for all tetracycline-resistance proteins that act by ribosomal protection. These proteins bind to the ribosome, hydrolyze GTP and cause release of tetracycline from the ribosome [67-69]. Antibiotic-free ribosomes able to translate mRNA are produced in this process.

We found Tet-coding genes in 20 genomes (Fig. 4, 5, 6). In one case (*Clostridium acetobutylicum*) two copies were found. The bacterial groups containing the Tet proteins include the producers of tetracyclines (*Streptomyces*), symbionts in the mammalian gut (*Lactobacillus, Bacteroides, Bifidobacterium*) and mammalian pathogens (*Bacillus, Staphylococcus, Streptococcus, Clostridium*). These are the groups most likely to have been in contact with tetracycline and have therefore acquired the resistance genes. The genes for Tet proteins are also present in the plant pathogen *Agrobacterium tumefaciens*. It is currently not clear why the genes have survived in the genome of this organism.

### LepA

The function of LepA in the cell is unclear. This protein, which was originally found in association with the cell membrane fraction, exhibits considerable similarity to the translation factor GTPases [70]. LepA crosslinks with ribosome-bound oxazolidinone antibiotics indicating that it can bind to the ribosome [71]. LepA has the unique property of back-translocating posttranslocational ribosomes [72]. The results suggest that it recognizes ribosomes after a defective translocation reaction and induces a back-translocation, thus giving EF-G a second chance to translocate the tRNAs correctly [72]. The gene has been inactivated in *Escherichia coli *[73] and *Staphylococcus aureus *[71]; the knockout strains are viable. It is therefore surprising to find that the presence of LepA coding genes in bacterial genomes is highly conserved and has very similar pattern to the IF2 genes: almost every genome has one copy (Figs. 4, 5, 6, 8). However, there are two exceptions: one of the sequenced strains of *Streptococcus pyogenes *has no LepA gene and *Pirellula *has two copies. The near-universal presence of LepA in bacteria suggests that this protein has an important function.

### Release factor 3

The first steps in the termination of translation utilize two types of release factor. Type I release factors (RF1 or RF2) recognize the termination codons and induce hydrolysis of the ester bond connecting the newly-made protein to the last tRNA [74,75]. The type II release factor (RF3) catalyses a GTPase-dependent release of the type I release factor from the ribosome [76,77].

It has been observed that an *Escherichia coli *strain with an inactivated RF3 gene is viable although its growth is disturbed [78,79]. This suggests that RF3 activity is not essential for the bacterial cell. This is in agreement with the present results showing that 119 of the 191 genomes analyzed contain the gene for RF3 but 72 do not (Figs. 4, 5, 6, 8). As expected, the gene is missing from most of the small genomes (*Mycoplasma, Chlamydia, Rickettsia, Wigglesworthia*), where only the core set of genes for the basic processes of gene expression have been preserved. In addition, several other groups of bacteria with large genomes contain no RF3 (*Bacillus, Mycobacterium, Streptomyces*).

In this context it is important to note that the GTPases involved in translation termination differ among the three superkingdoms. Bacterial release factor RF-3 is derived from the translocation factor EF-G family, whereas eukaryotic release factor eRF3 is a paralog of elongation factor EF-Tu/EF-1α; there is no corresponding release factor in Archaea [16,80]. It is currently not clear how the termination of translation works in organisms lacking RF3. The independence of the evolutionary origins of bacterial and eukaryotic RF3 suggests that loss of the gene could be compensated by duplication of a gene for another trGTPase, followed by diversion to take over the function of RF3. As in bacteria, the lack of RF3 does not correlate with the duplication of genes for other ribosome-associated GTPases (Figs. 4, 5, 6); our analysis does not support this scenario. Another possibility is suggested by the biochemical function of RF3 in the recycling of type I release factors: the weaker binding of type I release factors to the ribosome might compensate for the lack of RF3 function. The fact that weaker binding can compensate for the inactive GTPase has been demonstrated for a trGTPase: the eukaryotic homologue of IF2, eIF5B [81]. In the case of RF3, this prediction awaits experimental investigation.

### SelB

During synthesis of some proteins, co-translational incorporation of selenocysteine occurs [82]. It has been shown that specific UGA termination codons are used for selenocysteine insertion [82]. Incorporation of selenocysteine is directed by a specific RNA hairpin that follows the UGA codon [83-85]. This hairpin binds a ternary complex comprising translation factor SelB, GTP and selenocysteine-specific aminoacyl-tRNA [85-87]. In this way, selenocysteine-tRNA is directed to the ribosome containing a UGA codon in the A site.

*Bacillus subtilis *has no selenocysteine-specific tRNA or SelB protein [88]. Therefore, co-translational incorporation of selenocysteine does not occur in this organism. There is a high concentration of selenium in soil, the natural environment of *Bacillus subtilis*. It has been suggested that random incorporation of selenocysteine into proteins occurs in this organism because cysteinyl-tRNA synthetase cannot distinguish between cysteine and selenocysteine [88].

The distribution of the selenocysteine incorporation system in different bacteria has been analyzed previously [89]. SelB, analyzed in the current study, might be used as a marker for this system. Our analysis, in agreement with previous results [89], indicates that only 39 of the 191 genomes analyzed contain a gene for SelB (Figs. 4, 5, 6, 8). It is obvious that the lack of SelB is not confined to soil bacteria. In fact, many human symbionts and pathogens do not contain SelB. On the other hand, *Pseudomonas putida*, a soil bacterium, contains the gene.

Another surprising feature of the distribution of SelB is its sporadic presence in several bacterial groups. For example, *Clostridium perfringens *contains the gene but *Clostridium tetani *does not;*Treponema denticola *has the gene but *Treponema pallidum *does not; *Mycobacterium avium *has it and other *Mycobacteria *do not. It has been proposed [89] that this pattern is the result of two mechanisms, primarily speciation and differential gene loss, with some contribution from lateral gene transfer.

### TypA (BipA)

It has been shown that TypA regulates multiple cell surface and virulence-associated components in enteropathogenic *Escherichia coli *[90-92] and is required for growth at low temperatures [93]. In *Sinorhizobium meliloti*, TypA is required for growth under certain stress conditions [94]. Recently it has been proposed that TypA provides transcript-selective translational control [95]. It has been shown to function as a translation factor required specifically for expression of the global transcriptional modulator Fis [95]. It has been proposed that TypA destabilizes unusually strong interactions between the 5' untranslated region of *fis *mRNA and the ribosome [95]. It binds to ribosomes at a site coinciding with that for EF-G and has a GTPase activity that is sensitive to high GDP:GTP ratios and is stimulated by 70S ribosomes programmed with mRNA and aminoacylated tRNAs [95]. However, the molecular details of TypA action remain unknown.

Our analysis shows that 165 bacteria have one copy of a gene coding for TypA and 26 genomes have none (Figs. 4, 5, 6). The presence of this gene clearly correlates with genome size: it is present in all genomes larger than 2.8 Mb (Fig. 8). Indeed, if we exclude *Treponema denticola*, the largest genome lacking TypA is 1.5 Mb (Figs. 4, 5, 6). Genomes smaller than 1.5 Mb usually lack this gene.

### CysN/NodQ

In *Escherichia coli*, CysD and CysN are the two subunits of an ATP sulfurylase (ATPS) that produces adenosine-5'-phosphosulfate (APS) from ATP and sulfate, coupled with GTP hydrolysis. APS is then phosphorylated by an APS kinase, CysC, to produce 3'-phosphoadenosine-5'-phosphosulfate (PAPS), which is then used in amino acid biosynthesis [96]. In addition, *Sinorhizobium meliloti *(old name *Rhizobium meliloti*) appears to carry out the same chemistry for the sulfation of nodulation factors, oligosaccharides that are active in the roots of the host plant [97,98]. In *Sinorhizobium*, a heterodimeric complex comprising NodP and NodQ appears to possess ATP sulfurylase and APS kinase activities. Indeed, NodP shows strong amino acid sequence similarity to CysD, while NodQ appears to encode both CysN- and CysC-related sequences in a single ORF (the N and C termini of NodQ correspond to CysN and CysC, respectively) [98].

The gene for CysN/NodQ arose from an archaeal or eukaryotic elongation factor *1**α***(*EF-1**α***) by lateral gene transfer followed by a change in the function of the gene product [99]. The bacterial CysN has retained its GTPase activity that in this enzyme regulates production of APS. On the other hand it has lost the requirement for the ribosome to trigger its GTPase activity and probably has no function in translation [100].

Our analysis indicates that 74 genomes code for proteins of the CysN/NodQ subfamily (Figs. 4, 5, 6). In some genomes (*Nocardia, Sinorhizobium*) there are three genes for such proteins. We also used the APS kinase domain (CysC), absent from CysN but present in NodQ, to annotate the CysN and NodQ proteins separately. The NodQ coding gene was found in 21 genomes and the CysN coding gene in 56 (Figs. 4, 5, 6). Interestingly, *Nocardia *and *Sinorhizobium *have one gene for CysN and two genes for NodQ.

It is important to note that a phylogenetically-unrelated ATPS unable to hydrolyze GTP is present in many organisms [101,102]. As this protein family is present in all three domains of life we propose that it could be called ATPS1. Consistent with this proposal, the CysN/NodQ proteins that are present only in bacteria could be called ATPS2.

We have identified the genes coding for ATPS1 and marked them by asterisks in the ATPS column of Figs. 4, 5, 6. The results show that 106 genomes out of the 191 analyzed code for either CysN/NodQ or its functional analogue, ATPS1. The presence of either ATPS1 or ATPS2 (CysN/NodQ) mostly follows the phylogenetic grouping of bacteria: *Proteobacteria, Actinobacteria, Bacteroides *and *Spirochaetes *usually contain ATPS2 and *Bacilli, Cyanobacteria *and the *Thermus-Deinococcus *group contain ATPS1. The data also indicate that no gene for ATPS was identified in 85 genomes. It is currently not clear how sulfur assimilation occurs in these organisms.

## Conclusion

Our current understanding of the molecular mechanisms of trGTPases is based on studies using a very limited number of model organisms. The distribution of genes for trGTPase subfamilies in bacterial genomes suggests that there are considerable differences in the use of trGTPases in different bacteria. For example, RF3 has been considered a member of the "classical" set of trGTPases. It is now clear that many bacterial genomes do not code for this protein. On the other hand, LepA has been considered an obscure, auxiliary GTPase. The nearly ubiquitous presence of the gene for LepA in bacterial genomes calls for more attention to this protein. The unexpected divergence of the EF-G subfamily in many bacteria also points to a very exciting, still unanswered question.

## Methods

### Collection of sequences

The complete sequences of 191 bacterial genomes and annotated protein sequences were obtained from the RefSeq database [103] created on 10th of January, 2005. Additional unannotated genes were searched by running TBLASTN [104] against the intergenic regions of all 191 genomes with the following translational GTPases: IF-2, EF-Tu, SelB, EF-G, RF-3, TypA, LepA, CysN from *Escherichia coli *and Tet from *Bacillus cereus*. Matches with similarity more than 40% and match/query length ratios more than 70% were added, and thus the "updated protein database" was created.

The preliminary trGTPase dataset was obtained by running BLAST [104] against "updated protein database" with E-value cutoff 1 using *Escherichia coli *EF-Tu as a query. Multiple alignment was created by aligning all sequences against Hidden Markov Model GTP_EFTU from Pfam [105] using program HMMALIGN [18]. Unaligned ends and columns that contained more than 70% gaps were removed by the multiple sequence alignment editor BELVU [106]. Sequences with disrupted Walker A (G-1), Walker B (G-3) or guanine-specific binding domain (G-4) were rejected.

### Family-specific models

A phylogenetic tree was built using only the GTPase domain with the programs PROTDIST (JTT distances), NEIGHBOR and CONSENSE from the PHYLIP package [23]. Alternative trees for EF-G/Tet branch were drawn using TREEPUZZLE [24]. Trees were visualized using MEGA3 [107]. Nine clearly separated branches on the tree with high bootstrap values (> 85%) were used to build branch-specific HMMs. Sequences from each branch were aligned using CLUSTALW [108] and poorly-aligned ends were trimmed with BELVU [106]. From each branch-specific alignment a global HMM was built using HMMBUILD and calibrated using HMMCALIBRATE [18]. These HMMs were used for more specific searches and grouping of trGTPases with HMMSEARCH [18] against the "updated protein database". Searches were repeated at higher sensitivity levels until no more trGTPases were detected (Table 1).

To avoid artificial grouping of other GTPases into trGTPase subfamilies, thirty outgroup HMMs were built starting with 28 known TRAFAC GTPases, excluding trGTPases [16], CysC and CysD. Additional members of each outgroup were collected by running a BLAST search against "updated protein database" and keeping matches with E < 1e-40 and match length > 80% of query.

The APS kinase domain PF01583.8 from the Pfam database [109], absent from CysN and present in NodQ, was used to identify NodQ proteins. The ATP sulfurylase phylogenetically unrelated to CysN (ATPS1) was identified with Pfam domain PF01747.7 [101,102,105]. The IF2N domain was identified using Pfam domain PF04760 [39].

### Manual validation of trGTPase genes

We used a two-step decision scheme to eliminate protein sequences that cannot act as functional trGTPases. The first filter is based on minimal acceptable protein length (set at 2/3 of the average length of the members of a given subfamily) and integrity of the GTPase domain consensus elements (G1, G3, G4), which eliminates partial proteins (usually parts of pseudogenes annotated as ORFs) and proteins that are not GTPases [110]. We progressed further by analyzing why these seemingly non-functional proteins gave high scores in homology searches with HMMSEARCH. To analyze these cases at the genome level we used Artemis [111] and SHOWORF, PLOTORF and PRETTYPLOT from the EMBOSS package [112]. We found that in most cases a functional protein could be restored by an alternative gene start or frame-shift (17 cases), which we consider "restorable functionality". In only a few cases is there a partial gene in the genome (1) or genes inactivated by insertion (2 cases, 4 parts). Proteins with "restorable functionality" were added to the set of identified trGTPases.

### rRNA tree

To calculate a 16S rRNA-based phylogenetic tree, aligned rRNA sequences were obtained from the ribosomal RNA database RDP-II [113]. Columns with more than 80% gaps were removed from the alignment. The phylogenetic tree of ribosomal genes was calculated using fastDNAml [114].

## Authors' contributions

TM carried out the analysis and helped to draft the manuscript. MR and TT conceived of the study, and participated in its design and coordination and drafted the manuscript. All authors read and approved the final manuscript.

## Supplementary Material

Additional file 1 All cases of translational GTPases with "restorable functionality". Click here for file

Additional file 2 Full list of proteins found by the program HMMSEARCH with the threshold E-1. The first part contains a list of 1314 intact proteins; the second part contains a list of exceptions that were later included in the dataset (17 proteins); the third part contains a list of excluded proteins (5). Click here for file

## Figures and Tables

**Table 1:** The number of trGTPases identified on different E-value cutoffs

		**Number of GTPases found**									
		
**E-value**	**trGTP total**	**IF-2**	**EF-Tu**	**SelB**	**EF-G**	**Tet**^R^	**RF-3**	**TypA**	**LepA**	**CysN/NodQ**	**Out-group**
1.00E-200	1307	**191**	263	**36**	251	**20**	**118**	**161**	**190**	77	0
1.00E-100	1310	191	**265**	36	251	20	118	161	190	**78**	0
1.00E-10	1314	191	265	36	**255**	20	118	161	190	78	0
1	1314	191	265	36	255	20	118	161	190	78	0
10	1314	191	265	36	255	20	118	161	190	78	4

The number of trGTPases identified on different E-value cutoffs (first column) by HMMSEARCH [18]. Total numbers of trGTPases are shown in the second column. Proteins that did not pass the first validation criteria (intactness of the GTPase domain and length) are not included. The following nine columns list the numbers of trGTPases in different subfamilies. The "out-group" column shows the number of sequences identified by profiles based on GTPases of other families.
