# Supplementary material for: Phylogenetic distribution of translational GTPases in bacteria
Source: BMC Genomics. 2007 Jan 10;8:15. doi: 10.1186/1471-2164-8-15 (PMC1780047; doi:10.1186/1471-2164-8-15)
Supplement: Additional file 1 — All cases of translational GTPases with "restorable functionality". [file 1471-2164-8-15-S1.pdf]

Supplementary Material, Fig. S1. All cases of translational GTPases with "restorable functionality".

Supplement 1.

I Frameshifts

A. & **B.** SelB (*selB*) [YPO4052] from *Yersinia pestis CO92* and SelB [Yp3965] *Yersinia pestis biovar Mediavalis*

```
181 CATGAGAAGtttttggcgaacatgttggcggggggggggcggtatTGAtcacgctctgTT 240
  1           F  L  A  N  M  L  A  G  G  G  R  Y  *
 +1                                  G  G  G  G  I  D  H  A  L
```

C. RF-3 (*prfC*) [SO1210.1 & SO1211] *Shewanella onediensis*

```
801 GGAGTGGgctccaaaacgttgccccgtgaaagtgatctacgcaTGAtcatgccagatGAA 870
  1          A  P  K  R  C  P  V  K  V  I  Y  A  *
 -1              K  T  L  P  R  E  S  D  L  R  M  I  M  P  D
```

D. CysN (*cysN*) [BP0971] *Bordetella pertussis*

```
361 gccgccatcatcctggtcgacgcgacgcgacgcgggcggccgacgggcggctgctggccC 420
  1 A  A  I  I  L  V  D  A  T  R  R  G  R  P  T
 +5                   R  R  D  A  T  R  A  A  D  G  R  L  L  A
```

E. EF-Tu (*tufA* and *tufB*) [gi|27364609] in *Vibrio vulnificus CMCP6* *tufB* has C insertion in genome position 1182683

```
        1324113
tufA    ATCACAatctctacttctcacgta-gagtacgacactccagctcgccactacgcacacGT 1324171
        |||||||||||||||||||||||| |||||||||||||||||||||||||||||||||||
tufB181 ATCACAatctctacttctcacgtacgagtacgacactccagctcgccactacgcacacGT 240
      1         I  S  T  S  H  V  R  V  R  H  S  S  S  P  L
     +1                          E  Y  D  T  P  A  R  H  Y  A  H
```

F. EF-G (*fus*) *Shigella flexneri 2a* compared with *fus* gene from *2a 2457T* [*gi*|*30065374*] there is C insertion in the strain 2a 2457T

```
2a    1 atggctcgtacaacacccatcgcacg-ctaccgtaacatcggtatcagtgcgcacATCGA 60
        |||||||||||||||||||||||||| |||||||||||||||||||||||||||||||||
2457T 1 atggctcgtacaacacccatcgcacgcctaccgtaacatcggtatcagtgcgcacATCGA 60
      1 M  A  R  T  T  P  I  A  R  L  P  *
                                  Y  R  N  I  G  I  S  A  H
```

G. EF-G (*fus*) *Mycobacterium bovis fus2B* gene [gi|31791298] is compared with *fus* gene from *Mycobacterium tuberculosis*

```
                F  V  G  E  L  R  A  G  L  R  A  A  D  C  A  L  F  V  I  A
tubercu-        ttcgtgggtgagctgcgggccgggttgcgggccgccgattgcgcactgttcgtgatcgcg
losis           ||||||||||||||| || |||||||||||||||||||||||||||||||||||||||||
bovis       301 TTCGTGGGTgagctGTGg-ccgggttgcgggccgccgattgcgcactgttcGTGAtcgcg 359
              1          E  L  W  P  G  C  G  P  P  I  A  H  C  S  *
             -1              V  A  G  L  R  A  A  D  C  A  L  F  V  I  A
```

H. EF-G (*fus*) [N-term gi|27365097, C-term gi|27365098] *Vibrio vulnificus CMCP6*

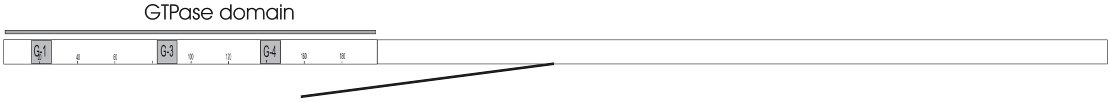


```
1051 ggccgtatggtggaaaATGcacgcaaacgaccgTAAcgaacttgaagcagctcaagcgggt 1111
   1 G  R  M  V  E  N  A  R  K  R  P  *
  +1                 M  H  A  N  D  R  N  E  L  E  A  A  Q  A  G
```

Supplement 1.

I. RF-3 (*prfC*) [gi|54307829] *Photobacterium profundum SS9*

```
 961 acttataccaaggcaTGAaaATGaaccatgtacgtattggtaaaaatgtcagtattTCTG 1020
   1 T  Y  T  K  A  *
  -1               M  K  M  N  H  V  R  I  G  K  N  V  S  I
```

J. TypA (*typA*) [gi|56752520] *Synechococcus elongatus PCC*

```
1081 cggggtgaactacactcggcattcTGAtcgaaacgATGcgtcgcgaaggttatgagttcC 1140
   1 R  G  E  L  H  S  A  F  *
  -1                                   M  R  R  E  G  Y  E  F
```

Supplement 1.

II Alternative start position

A. EF-Tu *(tuf)* [gi|29653575] *Coxiella burnetii*

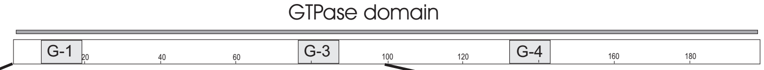


```
                                   -267      1
-294 ATGtcgaaggaaaaatttgtaagagag // atggacggagcgatattggtggtgagc 27
   1 M  S  K  E  K  F  V  R  E   // M  D  G  A  I  L  V  V  S   107
                             9      99
```

B. SelB (*selB*) [gi|54303209] *Photobacterium profundum SS9*

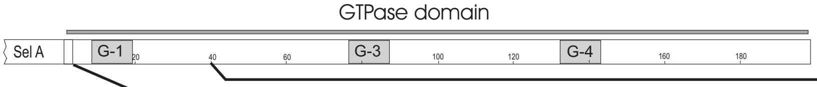


```
         A  A  I  *                                  -78   1
-120 ggctgcaatatgagttatgtcgtaggtactgctggccatgtcgat // atgactatcgatctc
              M  S  Y  V  V  G  T  A  G  H  V  D   // M  T  I  D  L
```

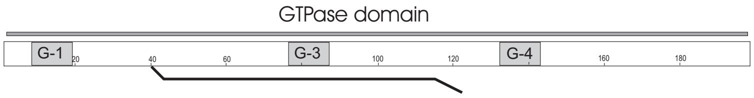

C. TypA (*typA*) [gi|32267271] *Helicobacter hepaticus*

```
                                  1
-123 atgcaaaatataagaaatattgctgtt // atggattctaatgatttagaaagagaa
   1 M  Q  N  I  R  N  I  A  V   // M  D  S  N  D  L  E  R  E
```

D. TypA (*typA*) [gi|21910714] *Streptococcus pyogenes*

```
                                  1
-136 atgactaacttaagaaacgatatccgt // atggattccaatgaccttgaaaaagaa
   1 M  T  N  L  R  N  D  I  R   // M  D  S  N  D  L  E  K  E
```

E. TypA (*typA*) [gi|21230395] *Xanthomonas campestris*

```
                                  1
-130 atgtctatcgaaaaactccgcaacatc // atggacagcaacgatcaggaaaaggaa
   1 M  S  I  E  K  L  R  N  I   // M  D  S  N  D  Q  E  K  E
```

F. LepA (*lepA*) [gil|15594434] *Borrelia_burgdorferi*

```
                                  1
-127 agtagtattagtattcgtaagaaaaac // atgcttgacagtatggatattgagcga
   1 S  S  I  S  I  R  K  K  N   // M  L  D  S  M  D  I  E  R
```

G. LepA (*lepA*) [gil|52081100] *Bacillus licheniformis ATCC 14580*

```
                                  1
-117 acgattacaaggcagtcgagaatccga // atgaaagaacagctcctcgactcaatg
   1 T  I  T  R  Q  S  R  I  R   // M  K  E  Q  L  L  D  S  M
```
